# Two randomized, controlled studies to assess the efficacy and safety of lotilaner (Credelio™) in preventing *Dermacentor reticulatus* transmission of *Babesia canis* to dogs

**DOI:** 10.1186/s13071-017-2473-1

**Published:** 2017-11-01

**Authors:** Daniela Cavalleri, Martin Murphy, Wolfgang Seewald, Jason Drake, Steve Nanchen

**Affiliations:** 1 Elanco Animal Health, Mattenstrasse 24a, CH-4058 Basel, Switzerland; 2 Elanco Animal Health, 2500 Innovation Way, Greenfield, IN 46140 USA

**Keywords:** Lotilaner, Credelio, *Dermacentor reticulatus*, *Babesia canis*, Dog, Efficacy

## Abstract

**Background:**

Dogs worldwide are at risk of *Babesia* spp. infections. Preventive efficacy of lotilaner tablets (Credelio™, Elanco) against *Babesia canis* was evaluated in two studies.

**Methods:**

Sixteen dogs in Study 1 and 12 dogs in Study 2, all seronegative and polymerase chain reaction (PCR) negative for *B. canis*, were randomized to a sham-treated control group or a lotilaner (20–43 mg/kg) treatment group, administered on Day 0 (Study 1: *n* = 8/group; Study 2: *n* = 6/group). Dogs were each infested with 50 *Dermacentor reticulatus*, a percentage of which (Study 1: 8.0–30.0%; Study 2: 12.2%) were infected with *B. canis*, in Study 1 on Days 2, 7, 14, 21 and 28, and in Study 2 on Day 28. Ticks were removed and counted on Day 30 in Study 1, and Day 34 in Study 2. Blood was collected for *Babesia* detection via smear, PCR and immunofluorescence assay (IFA) in Study 1 on Day 2, then approximately weekly through Day 56, and in Study 2 at weekly intervals between Days 28 to 49, and on Days 63 and 91. Additional samples were collected from dogs with body temperature > 39.4 °C (measured three times weekly, from Days 7 to 56 in Study 1 and from Days 35 to 56 in Study 2) and positive for *B. canis* on blood smear. Dogs with confirmed infections were rescue-treated, removed from the study and, in Study 1, replaced.

**Results:**

Across both studies *B. canis* infection of ticks ranged between 8.0–30.0%. In Study 1, all control dogs were positive for *B. canis* on blood smear and PCR on Day 10 and IFA on Day 21; on Day 21 seven of eight replacement control dogs were *B. canis*-positive; no replacement dogs were *B. canis-*positive following tick removal on Day 30. In Study 2, all control dogs were *B. canis-*positive on Day 56. All lotilaner-treated dogs remained *B. canis*-negative at all assessments in both studies.

**Conclusion:**

Lotilaner efficacy was 100% in preventing establishment of *B. canis* infection, despite post-treatment challenge with infected ticks on Days 2, 7, 14, 21 and 28.

## Background

Canine babesiosis is an important vector-borne disease which occurs throughout the world, being transmitted by ixodid ticks including *Rhipicephalus*, *Dermacentor* and *Haemaphysalis* species [1, 2]. In Europe, the occurrence and spread of infection of dogs with *Babesia canis* is linked to the geographical distribution of the ornate dog tick, *Dermacentor reticulatus* [1]. Favouring cool and wet climates, the distribution of *D. reticulatus* ranges from northern Portugal through the British Isles and Baltic countries into eastern Europe, including Balkan countries, and Russia [2–5]. Dogs in those widespread regions are therefore at risk of contracting the infection, which can cause intravascular and extravascular hemolysis, resulting in anemia and thrombocytopenia, and clinical signs such as fever, lethargy and anorexia [1]. Demonstration that an acaricide with efficacy against *D. reticulatus* acts with sufficient speed to prevent transmission of *B. canis* is an important consideration for optimizing protection of dogs against these pathogenic effects.

The emergence of the isoxazoline family of compounds promises to transform the control of insect and acarine infestations in dogs, adding the oral administration route as an option for tick control. The most recent member of this family to receive regulatory approval is lotilaner, formulated in a flavoured chewable tablet (Credelio™, Elanco). Laboratory and field studies demonstrated that lotilaner, at the approved dose rate of 20–43 mg/kg, has potent activity against fleas and ticks and is effective in the treatment of demodectic mange [6–10]. At the label dose rate, lotilaner has been shown to have a very wide safety margin, including when used in young puppies [11]. Lotilaner’s effects on fleas have been evident from 2 h post-treatment, and against the tick *Ixodes ricinus* from 4 h after treatment [6, 7]. Observations of the rapid onset and sustained high efficacy of lotilaner against ticks led to the hypothesis that treatment of dogs would be effective in preventing the transmission of tick-borne pathogens. To test that hypothesis, an investigation was undertaken in which dogs were challenged with infestations of *B. canis*-infected *D. reticulatus*.

## Methods

Two assessor-blinded, randomized, negative-controlled, laboratory studies investigated the efficacy of lotilaner in preventing *D. reticulatus* transmission of *B. canis* to dogs. Studies were performed in compliance with the principles of Good Clinical Practice [12, 13]. With the exception of the Dispenser, all personnel carrying out study activities (e.g. general health observations, clinical observations, animal weighing, animal maintenance, preparation of ticks, infestations with ticks, tick counts, physical examination, safety data reviewing) were blinded to the treatment administered to each dog. The investigator in both studies was not blinded in order to oversee the replacement of *Babesia*-infected animals.

### Animals

In Study 1, 24 dogs were acclimatized to facility conditions from Day -7 to Day -1. A veterinary examination (Day -7), general health observations (all days) and weighing of all dogs (Day -7 and Day -1) were performed during acclimatization. An additional eight replacement dogs meeting the inclusion criteria and no exclusion criteria, were acclimatized to the study facility from Day 15 to Day 27. The 32 dogs, males and females, that were included throughout the study consisted of 31 purpose-bred cross-breed dogs and one Beagle. Ages ranged from 11 months to 6 years and 7 months, and dogs weighed between 10.4–18.8 kg.

When the results (serology, PCR and blood smear) of Study 1 became available, it was clear that the control dogs challenged on Day 27 of Study 1 did not show any evidence of *Babesia* infection that followed this challenge. As it was not possible to re-challenge the dogs at this time and in order to confirm the efficacy of Lotilaner in preventing transmission of *B. canis* at the end of the planned protection period, a follow-on study was designed to provide these additional data. In Study 2, 12 dogs were acclimatized to facility conditions from Days -3 to -1. There were five purpose-bred cross-breed dogs and seven Beagles, male and female, aged from 9 months to 5 years and weighing 10.6–17.3 kg. A veterinary examination (Day -3), general health observations (all days) and weighing of all dogs (Day -3 and Day -1) were performed during acclimatization.

For inclusion in either study, dogs were required to be clinically healthy and non-pregnant, at least 7 months of age, to weigh no more than 22 kg, to have an appropriate temperament to allow handling for study procedures and to be infested with at least 13 live, attached ticks (corresponding to a retention rate of 25%, as confirmation of the viability of the ticks and host suitability), 48 h following a challenge of 50 *D. reticulatus* during the week immediately prior to the day of treatment*.* The ticks used for this challenge were not infected with *B. canis*. Dogs could not have been treated with a topical or systemic acaricide/insecticide during the 12 weeks preceding Day 0 (6 months for isoxazolines) and had to be sero-negative and polymerase chain reaction (PCR) negative for *B. canis* prior to Day 0*.* Dogs were identified by electronic transponders with unique alphanumeric codes, and were maintained in concrete-floored cages, approximately 2.0 × 3.0 m., that were part of an environmentally controlled indoor animal unit. Each cage was fitted with a sleeping bench. At least one toy/chew was made available to each dog (replenished weekly). While no physical contact between dogs was possible, they had visual and auditory contact with conspecifics. All dogs were allowed at least 20 min of exercising twice a week, outside the times when they were infested with ticks. A photoperiod of 12 h light: 12 h darkness was maintained, with lighting provided by overhead fluorescent lamps.

All dogs had been dewormed prior to study initiation and did not harbor resident tick or flea infestations. During acclimatization and throughout each study, the dogs were fed once daily with an age-appropriate commercial dry-food diet according to the food manufacturer’s recommendation. Food and water were provided in stainless steel bowls and the water was replenished at least twice daily.

In Study 2, the dogs were moved on Day 38 to outside housing facilities exposed to ambient temperatures and photoperiod, for logistical reasons. These outdoor cages each had an indoor area with sleeping kennels and an outside run area. The dimensions of these cages were at least 4.5 m^2^ roofed area with a run area of at least 18 m^2^. Dogs were individually or communally housed during this period, within their specific study groups. The change had no impact on animal welfare and was approved by the IACUC (Institutional Animal Care and Use Committee) of the study site.

### Randomization and treatment

Each study was comprised of two treatment groups. Group 1 dogs were sham-treated, and Group 2 dogs received flavoured chewable tablets.

In Study 1, the eight dogs with the lowest live attached tick counts on Day -4 (12–24 live attached ticks, 48 ± 4 h post-infestation) were excluded. The 16 included dogs were ranked in descending order of live attached tick counts and blocked into eight blocks of two. Within blocks, dogs were randomly allocated to a study group. The eight dogs that were excluded after the treatment randomization remained in the study facility as replacements for any randomized dogs that were diagnosed as being infected with *B. canis.*

In Study 2, the 11 dogs meeting the inclusion criterion of tick retention (at least 13 live attached ticks, 48 h following the Day -3 infestation) were ranked in descending order of Day -1 counts and blocked into five blocks of two and one block of one. Within the five blocks, dogs were randomly allocated to a study group and the one-dog block was assigned to the lotilaner group. An additional dog meeting the inclusion criteria and no exclusion criteria was included as the sixth control group dog on Day 25.

In both studies, all dogs assigned to Group 2 were treated on Day 0. Treatment was available in strengths of 56.25, 112.5, 225 and 450 mg lotilaner, and tablets were administered whole.

Thirty minutes (± 5 min) prior to the scheduled treatment/sham treatment, each dog was offered half of the daily ration of wet food at the recommended rate. At least two-thirds of the ration of wet food offered (corresponding to one third of the full daily ration) had to be consumed prior to treatment (one dog in Study 1 and three dogs in Study 2 consumed less food than intended). Sham treatment of the dogs in the negative control group involved removal from their cages and placement on the dosing table.

Specific vomit checks were performed immediately after lotilaner administration and at approximately 30 min and 1 h later. Observations for adverse events (AEs) were completed at approximately 1, 6 and 8 h after administration.

### Tick infestations and counts

For challenges using a laboratory-bred strain of *D. reticulatus* each dog was placed in an infestation chamber (70 × 80 × 90 cm) and manually restrained for 10 min to facilitate tick attachment. Dogs remained in the chamber for 4 h after application of ticks.

After allocation to groups, dogs meeting the inclusion criteria and no exclusion criteria were infested on challenge days with approximately 50 (± 4) viable, adult, unfed *B. canis*-infected *D. reticulatus* ticks (50% female: 50% male approximately). In Study 1 challenges were completed on Days 2, 7, 14, 21 and 28, and in Study 2 only on Day 28. A sample of 50 ticks used for each infestation was tested by PCR analysis to verify infectivity.

Ticks were found through direct observation by parting of the hair coat and palpation per the standard facility procedure. In Study 1, ticks were removed from any dog diagnosed with *Babesia* infection, and infected dogs were discontinued from the study. Replacement dogs were then included for control-group challenges prior to the next tick challenge, on Days 13 and 27. Ticks were counted in situ (without removal) on Days 4, 9, 16 and 23 (48 ± 4 h after each infestation). The final tick removal and count for all dogs in Study 1 was performed on Day 30, and in Study 2 ticks were removed from the dogs on Day 34 and counted. Tick counts on any replacement dog were included for subsequent tick efficacy assessments.

### *Babesia* infection determination

Venous blood samples of at least 3 ml were collected into EDTA tubes from all dogs for PCR analyses, prior to any infected-tick infestation, in Study 1 on Days -7, 2, 14, 21, 28, 35, 42, 50 and 56, and in Study 2 on Days -3 (Day 22 for the additional dog), 28, 35, 42, 49, 63 and 91. Additional samples were collected from any dog with suspected *Babesia* infection (body temperature > 39.4 °C, on Days 8, 10, 13, 24, 27, 31, 34, 38 and 45 in Study 1 and 44, 46, 51 and 53 in Study 2) and confirmed positive for *B. canis* on blood smear, prior to rescue treatment. Blood samples were also collected into plain tubes for serum analyses at the same time-points. Approximately 1 ml of blood was taken from the 3 ml whole-blood sample and stored in a cryo-tube in a -80 °C freezer (< -70 °C), which served as a reserve sample for PCR analysis. The remaining whole blood samples were maintained at ambient conditions and transferred to the laboratory for analysis.

Serum was recovered from the plain tubes and divided into primary and duplicate aliquots. Duplicate aliquots were frozen at ≤ -35 °C on the day of collection. Primary aliquots were stored at 2–8 °C until assayed for *B. canis* antibodies using the immunofluorescence antibody (IFA) assay (MegaFLUO® BABESIA canis).

Total genomic DNA was isolated from whole blood samples, using a commercial genomic DNA isolation kit (GeneJET Genomic DNA Purification Kit, Thermo Scientific, Vilnius, Lithuania). Polymerase chain reaction (PCR) entailed the use of primers specific to a region of the *B. canis* rDNA [14]. Up to 400 ng isolated DNA served as template for PCR amplification of the target region. PCR products were analyzed using agarose gel electrophoresis and results documented. A PCR product of approximately 302 bp indicated the presence of the *B. canis* rDNA target region in the sample. Positive, negative, no template, as well as internal amplification controls, were included in each run.

Any dog with confirmed *B. canis* infection was removed from the study, rescue-treated and replaced with a replacement dog prior to the next tick challenge day. Rescue treatment consisted of diminazine [Berenil® RTU, manufactured by MSD Animal Health, Boxmeer, The Netherlands (1 ml/20 kg, intramuscular injection)] followed the next day by imidocarb [Forray® 65, Manufactured by Schering-Plough Animal Health, Friesoythe, Germany (1.2 ml/20 kg, subcutaneous injection)]. In Study 1, eight replacement dogs were initially available for replacement, and subsequently an additional eight animals were enrolled as replacements on Day 15.

### Health assessments

Health observations of all dogs were made at least once daily. All dogs were examined under veterinary supervision, pre-inclusion and at approximately weekly intervals in Study 1 from Day 7 and in Study 2 from Day 28 until the end of each study.

Examinations included blood collections for PCR and IFA assays from all dogs at regular intervals. In Study 1, dogs were weighed on Days -7, -1 and 56 or 57, and in Study 2 on Days -3, 22 (for an additional dog included), 28 and 56. Rectal body temperatures were recorded three times weekly from Day 7 in Study 1 and Day 35 in Study 2 to Day 56 in both studies. In addition to the scheduled PCR and IFA assays, blood smears were evaluated for *B. canis* merozoites from any dog with abnormally high body temperature (> 39.4 °C) or clinical signs of babesiosis. If a dog was positive on blood smear for *B. canis*, blood was collected for PCR and IFA analysis.

Efficacy was determined by the number of dogs diagnosed as being infected with *B. canis* in the untreated control and treated groups. Infection was confirmed if a blood smear was positive for *B. canis*, and if blood samples were positive on both IFA test and PCR analysis.

### Statistical analysis

The *B. canis* prevention efficacy for lotilaner was calculated as follows:

$$ \mathrm{Efficacy}\left(\%\right)=100\times \left(\mathrm{Pc}-\mathrm{Pt}\right)/\mathrm{Pc} $$

where Pc is the percent of untreated control dogs (Group 1) diagnosed as infected with *Babesia* at any point and Pt is the percent of dogs in the lotilaner group diagnosed as infected (Group 2).

The rate of *Babesia* infection was compared between groups with the Fisher’s exact test. Tick counts were compared between groups using an ANOVA (Proc GLM procedure in SAS) with a treatment effect, both on the original scale and after applying a logarithmic transformation on the tick (count +1) data. The level of significance of the formal tests was set at 5% (i.e. *P*-value < 0.05), all tests were two-sided.

## Results and discussion

In Study 1, the dose rates of lotilaner ranged between 20.8–35.2 mg/kg, and in Study 2 between 26.0–40.1 mg/kg. None of the lotilaner-treated dogs in either study vomited following dose administration and there were no treatment-related AEs.

For the Day 2 challenges, the 30% *B. canis* infection rate of ticks aligned with an earlier report that described an infection rate of 33% [15], while in different reports, the infection rate ranged from 8.0 to 11.8% [13, 14, 16]. The lower rate reported from those studies is also consistent with the other tick infection rates in Study 1 and the rate in Study 2 (Table 1).

**Table 1:** Percentage of ticks infected with *Babesia canis* at each challenge, as determined by polymerase chain reaction

	Study 1					Study 2
Days after treatment	2	7	14	21	28	28
Percent infected	30.0	10.4	11.8	8.3	8.0	12.2

Consistent with earlier studies assessing the efficacy of lotilaner against *D. reticulatus* and other species, the tick counts throughout Study 1 and the count in Study 2 demonstrated efficacy of 99.1–100% for the challenges (Table 2) during the month following treatment [9, 17].

**Table 2:** Geometric (arithmetic) mean counts of live *Dermacentor reticulatus* and percent efficacy

Day	Untreated control		Lotilaner			Comparison
	Mean	Range	Mean	Range	Efficacy (%)	
Study 1						
4	22.2 (22.4)	18–29	0.2 (0.3)	0–1	99.1 (98.7)	*t*_(14)_ = 24.07, *P* < 0.0001
9	37.6 (39.1)	23–52	0.2 (0.3)	0–2	99.5 (99.2)	*t*_(14)_ = 20.16, *P* < 0.0001
16	13.3 (14.3)	5–19	0.0 (0.0)	0–0	100 (100)	*t*_(14)_ = 18.54, *P* < 0.0001
23	25.1 (32.5)	4–87	0.0 (0.0)	0–0	100 (100)	*t*_(14)_ = 11.26, *P* < 0.0001
30	23.4 (25.1)	12–41	0.0 (0.0)	0–0	100 (100)	*t*_(14)_ = 21.71, *P* < 0.0001
Study 2						
34	23.7 (24.5)	15–36	0.0 (0.0)	0–0	100 (100)	*t*_(10)_ = 28.45, *P* < 0.0001

No infections with *B. canis* were detected by blood smear, IFA or PCR in any lotilaner-treated dog at any point during either study. Under the study challenge conditions, on Day 10 all eight control dogs were positive for *B. canis* on blood smear, IFA test and PCR analysis, indicating that the Day 2 challenge was successful in transmitting the pathogen (Tables 3 and 4). Elevated body temperatures (> 39.4 °C) were observed by Day 10 in all the control dogs, with maximum temperatures ranging between 39.7–40.9 °C. Moderate clinical signs consistent with *B. canis*-induced intravascular haemolysis, that were observed on Day 10, included pale mucous membranes and lethargy, and haemoglobinuria was observed in one of these dogs. All eight control dogs were removed from the study and subsequently recovered following rescue treatment.

**Table 3:** Summary of assessments for *Babesia canis* infection

Test	Test day	Cumulative proportion positive		Efficacy (%)	Comparison
		Control	Lotilaner		
Blood smear	2	8/8	0/8	100	*χ* ^2^_(1)_ = 16, *P* = 0.0002
	14	15/16	0/8	100	*χ* ^2^_(1)_ = 20, *P* < 0.0001
Study 1	28	15/23	0/8	100	*χ* ^2^_(1)_ = 10.11, *P* = 0.0024
Study 2	28	6/6	0/6	100	*χ* ^2^_(1)_ = 12, *P* = 0.0022
IFA	2	8/8	0/8	100	*χ* ^2^_(1)_ = 16, *P* = 0.0002
	14	15/16	0/8	100	*χ* ^2^_(1)_ = 20, *P* < 0.0001
Study 1	28	15/23	0/8	100	*χ* ^2^_(1)_ = 10.11, *P* = 0.0024
Study 2	28	6/6	0/6	100	*χ* ^2^_(1)_ = 12, *P* = 0.0022
PCR	2	8/8	0/8	100	*χ* ^2^_(1)_ = 16, *P* = 0.0002
	14	15/16	0/8	100	*χ* ^2^_(1)_ = 20, *P* < 0.0001
Study 1	28	15/23	0/8	100	*χ* ^2^_(1)_ = 10.11, *P* = 0.0024
Study 2	28	6/6	0/6	100	*χ* ^2^_(1)_ = 12, *P* = 0.0022

*Abbreviations*: *IFA* immunofluorescence assay, *PCR* polymerase chain reaction

**Table 4:** Number of days to a positive result (control dogs only; no positive results in treated dogs)

	Test	First infestation day	Proportion positive	Mean days post-challenge to positive test (SD)
Blood smear	Study 1	2	8/8	8.0 (0.0)
		14	7/8	8.7 (1.0)
		28	0/8	None positive
	Study 2	28	6/6	8.0 (0.0)
IFA	Study 1	2	8/8	19.0 (0.0)
		14	7/8	16.0 (3.4)
		28	0/8	None positive
	Study 2	28	6/6	26.8 (2.9)
PCR	Study 1	2	8/8	8.0 (0.0)
		14	7/8	7.0 (0.0)
		28	0/8	None positive
	Study 2	28	6/6	7.0 (0.0)

*Abbreviation*: SD, standard deviation

Following the challenges on Day 14 and Day 21, seven of the eight replacement control dogs were positive for *B. canis* infection by PCR on Day 21, by blood smear between Days 21 and 24 inclusive, and by IFA test on Day 21 (Table 3). None of these dogs became hyperthermic, five showed moderate signs of babesiosis that included pale mucous membranes and lethargy, and in three dogs haemoglobinuria was observed. The seven *B. canis*-positive control dogs were removed from the study and subsequently recovered following rescue treatment. The one control dog that did not show evidence of *B. canis* infection had only four live ticks identified on Day 23, despite infestations being placed on Days 14 and 21. At this point live tick counts in the other control group dogs, from the same infestation days, ranged between 17 and 87. The relatively low number of ticks infesting this dog provides a likely explanation for the failure of *B. canis* transmission. This dog was retained and then included in the Day 28 challenge.

In Study 1 the final tick challenge was completed on Day 28 and ticks were removed on Day 30. All control dogs (the seven replacement dogs plus the control dog not infected at Day 21) and all lotilaner-treated dogs receiving this challenge remained negative for *B. canis* at all assessments through Day 56, and none showed any clinical evidence of infection. The failure of any of these control dogs to develop *B. canis* infection was attributed to the removal of ticks at 48 h post-challenge, apparently before there was adequate opportunity for pathogen transmission. These laboratory findings align with earlier work, indicating that sporozoites of *Babesia* spp. are not transmitted from the salivary glands until at least 48 h after tick attachment [18].

Because lotilaner has been shown to provide a high level of efficacy against *D. reticulatus* for at least 1 month post-treatment, it was considered relevant to investigate whether dogs would be protected against a Day 28 challenge with *B. canis*-infected ticks. Therefore, Study 2 was initiated with a challenge only on Day 28 post treatment. As in Study 1, tick counts conducted after this challenge demonstrated lotilaner efficacy of 100% (Table 2). No infections with *B. canis* were detected by blood smear, IFA or PCR in any lotilaner-treated dog, while all six untreated control dogs were positive on each test by Day 56 (Tables 3 and 4).

## Conclusion

Lotilaner was safe and efficacy was 100% in preventing the establishment of *B. canis* infection, despite challenge with infected ticks on Days 2, 7, 14, 21 and 28 after treatment.

## Abbreviations

AE: Adverse event
ANOVA: Analysis of variance
EDTA: Ethylenediaminetetraacetate
IFA: Immunofluorescence assay
PCR: Polymerase chain reaction
